# Chitosan-assisted differentiation of porcine adipose tissue-derived stem cells into glucose-responsive insulin-secreting clusters

**DOI:** 10.1371/journal.pone.0172922

**Published:** 2017-03-02

**Authors:** Hui-Yu Liu, Chih-Chien Chen, Yuan-Yu Lin, Yu-Jen Chen, Bing-Hsien Liu, Shiu-Chung Wong, Cheng-Yu Wu, Yun-Tsui Chang, Han-Yi E. Chou, Shih-Torng Ding

**Affiliations:** 1 Department of Animal Science and Technology, National Taiwan University, Taipei City, Taiwan, R.O.C.; 2 Institute of Biotechnology, National Taiwan University, Taipei City, Taiwan; 3 Graduate Institute of Oral Biology, National Taiwan University, Taipei City, Taiwan; Instituto Butantan, BRAZIL

## Abstract

The unique advantage of easy access and abundance make the adipose-derived stem cells (ADSCs) a promising system of multipotent cells for transplantation and regenerative medicine. Among the available sources, porcine ADSCs (pADSCs) deserve especial attention due to the close resemblance of human and porcine physiology, as well as for the upcoming availability of humanized porcine models. Here, we report on the isolation and conversion of pADSCs into glucose-responsive insulin-secreting cells. We used the stromal-vascular fraction of the dorsal subcutaneous adipose from 9-day-old male piglets to isolate pADSCs, and subjected the cells to an induction scheme for differentiation on chitosan-coated plates. This one-step procedure promoted differentiation of pADSCs into pancreatic islet-like clusters (PILC) that are characterized by the expression of a repertoire of pancreatic proteins, including pancreatic and duodenal homeobox (Pdx-1), insulin gene enhancer protein (ISL-1) and insulin. Upon glucose challenge, these PILC secreted high amounts of insulin in a dose-dependent manner. Our data also suggest that chitosan plays roles not only to enhance the differentiation potential of pADSCs, but also to increase the glucose responsiveness of PILCs. Our novel approach is, therefore, of great potential for transplantation-based amelioration of type 1 diabetes.

## Introduction

Type 1 diabetes mellitus (T1DM) is a disease of impaired glucose homeostasis resulting from autoimmune destruction of pancreatic islet cells accounting for 5–10% of diabetes mellitus worldwide [1]. Insulin replacement has been the primary treatment. However, this treatment does not mimic the full glycemic control of functional β-cells, and presents potential complications and compromises quality of life with daily injections [2]. Approaches using tissue transplantation with human or animal pancreatic islets [3–6] and cell therapy [7] with various sources of β-cell-like cells have emerged as alternatives. These cells can be derived from embryonic [8,9] or adult-tissue stem cells obtained from liver [10], umbilical cord blood [11,12], skin fibroblasts [13], placenta [14], bone marrow [15], pancreatic progenitor cells [16] and periosteum [17].

Compared to other stem cell sources, adipose tissue-derived stem cells (ADSC) possess the unique advantage of being easily and repeatedly accessible with abundance, and their robust multipotency has been demonstrated to be a most promising source for cell therapy and regenerative medicine [3,18–20]. For instance, human and murine ADSCs have been isolated from fat depots and differentiated into glucose-responsive insulin-secreting clusters, showing encouraging effects on amelioration of T1DM in animal models [21–25]. These research models provide the concept for treating diabetes mellitus with *ex vivo* generated insulin-secreting cells [24,25]. Moreover, our group has verified the similarities and differences of porcine ADSCs and bone marrow mesenchymal stem cells (BMMSCs). In a proliferation assay, porcine ADCSs showed higher colony forming unit fibroblasts than BMMSCs and surface marker assays indicated that both BMMSCs and ADSCs are positive for CD29 and CD44 mesenchymal stem cell surface markers and negative for CD31 and CD45 hematopoietic cell markers. A wound healing assay revealed that ADSCs migrated faster than BMMSCs (data not published).

However, the vast dissimilarities between rodents and humans, such as the immune system, many aspects of metabolism, anatomy, body size and natural life span, have been of primary concern to the development of transplantation medicine derived from murine models. Hence, translational studies conducted in large animals are essential and critical for the eventual development of transplantation medicine [26]. Among various options, pigs present close resemblance in anatomical and physiological composition to humans, and genetic manipulation to produce ‘humanized pigs’ is under way to serve as a promising source of materials for transplantation medicine.

Our research group has been using ADSC from humans, mice and pigs to study the regulation of adipogenesis and lipid metabolism [27–29]. In the present study, we present a protocol to obtain ADSC from porcine fat depots and trigger its differentiation to the formation of pancreatic islet-like cells (PILC). In addition to the aforementioned advantages of robustness and easy access offered by porcine ADSCs, our method is devoid of further genetic modifications to the cells that may bring concerns of uncontrolled tumorigenicity. Furthermore, we introduce the use of chitosan as a biocompatible scaffold to greatly enhance the formation of cell clusters with insulin secretion. Chitosan is a natural polysaccharide derived from the shells of crustaceans (such as crabs, shrimp, and lobsters), and has been claimed to provide an ideal scaffold for the culture of cells without toxic and immunogenic effects. Chitosan has been shown to promote cell adhesion, spheroid formation and differentiation of stem cells. [30–32]. Herein we demonstrated that, with the assistance of a chitosan matrix, ADSCs derived from dorsal subcutaneous adipose tissues of piglets can be differentiated into high insulin-secreting PILC that are responsive to a glucose challenge.

## Materials and methods

### Isolation of ADSC from pig subcutaneous fat depots

9-day-old piglets (Landrace x Yorkshire) of 3 to 4.5 kg bodyweight were purchased from a local farm, sacrificed by electric shock and exsanguinated according to the procedures approved by the Animal Care and Use Committee of National Taiwan University. The dorsal subcutaneous fat tissue was surgically dissected and processed as previously described [27]. In brief, 10 g of adipose tissue slices were minced and then digested with 6000 IU (0.6 mg / mL) of collagenase (Sigma C6885, Sigma-Aldrich, St. Louis, MO, USA) in sterile Krebs Ringer bicarbonate buffer containing glucose (25 mM) at 37°C for 90 min and filtered through a nylon mesh (100 μm opening). The stromal vascular fraction (SVF) was isolated by centrifugation at 800 × *g* for 10 min and washed three times in suspension with Dulbecco’s modified Eagle medium (DMEM catalogue #12800–017, Gibco) containing penicillin (100 U /ml), streptomycin (100 mg /mL) and amphotericin B (1.5 μg/ml, Caisson Laboratories, North Logan, UT, USA). Prior to the last wash, SVF were treated with red-blood-cell-lysing buffer (155 mM NH_4_Cl, 5.7 mM K_2_HPO_4_, and 0.1 mM EDTA at pH 7.3) for 10 minutes to remove the red blood cells. The washed SVF were then suspended in σ-MEM (catalogue # M0894, Sigma-Aldrich) containing 10% fetal bovine serum (FBS; Biological Industries, Kibbutz Beit Haemek, Israel) and penicillin, streptomycin and amphotericin B (Caisson Laboratories, North Logan, UT, USA) and plated at a density of 6 × 10^4^ cells/cm^2^ on chitosan-coated (see below for preparation) or regular dishes. Cells were cultured at 37°C in air containing 5% CO_2_ for 48 hours with medium changed to allow for full attachment.

### Flow cytometry analysis

To analyze the surface markers of pADSCs by flow cytometry, cells were harvested with 100 μL of 0.25% trypsin-EDTA for 5 min at 37°C and washed twice in washing buffer (49.5 mL PBS and 0.5 mL FBS) by centrifugation at 400 × *g*. Cells (1 x 10^5^/100 μL) were incubated at 4°C for 30 min (covered with foil) with antibodies against phycoerythrin (PE)-conjugated CD29 (CD29-PE), CD31-PE, CD44-PE, CD45-PE, MHC I-PE and MHC II-PE (Sigma-Aldrich, St. Louis, MO, USA) for direct staining. The reaction was stopped by washing twice in 500 μL of washing buffer with 5-min 700-× *g* centrifugation, then fixed in fixing buffer (48.1 ml PBS + 0.5 mL FBS +1.3 mL of 37% formaldehyde) for flow cytometry analysis in the FACSCalibur system (BD Biosciences, New Jersey, USA) using CellQuest^™^ software (Becton Dickinson, San Jose, CA, USA).

### Differentiation of porcine (p)ADSC into adipocytes, osteocytes and chondrocytes

The multipotency of pADSC was evaluated by differentiating cells into adipocytes, osteocytes and chondrocytes according to procedures previously described [28]. After removing the maintenance medium, the confluent pADSC were incubated with differentiation medium. For adipocytes, differentiation medium was DMEM/F12 containing 10% FBS, 1 μM dexamethasone, 0.5 mM methyl-isobutylxathine, 10 μg / mL insulin and 100 mM indomethacin and for osteocytes, it was DMEM/F12 containing 10% FBS, 1 mM dexamethasone, 10 mM β-glycerophosphate and 50 μm ascorbate-2-phosphate. Cells were cultured at 37°C in air containing 5% CO_2_ with medium changed every three days. For chondrocyte differentiation, cells were trypsinized and 2.5 × 10^5^ cells were centrifuged at 150 × *g* for 5 min at room temperature in 15-mL conical polypropylene tubes. The culture medium was then replaced with 1 mL of chondrogenic differentiation medium consisting of αMEM, 1% FBS, 6.25 μg/mL insulin, 50 μM ascorbic acid, and 10 ng/mL transforming growth factor-β1 (R&D Systems, Minneapolis, MN). Plates were cultured at 37°C in air containing 5% CO_2_ with medium changed every three days. Unless indicated otherwise, all reagents used for cell culture were from Sigma-Aldrich.

Oil red O staining [33] for differentiated adipocytes at day 6 was performed by initially fixing cells in 10% formaldehyde for 10 min. After two PBS washes, the formaldehyde was removed and adding 100% propylene glycol for 1 min, followed by Oil red O solution (Sigma-Aldrich) at room temperature for 15 minutes. After replacing with 60% propylene glycol for 1 minute, the residual oil red O was removed with water for light microscopy. For alizarin red S staining [34] of osteocytes, the medium was removed completely, cells were fixed in neutrally buffered 10% formalin and the formalin was removed with three distilled water washes. Two % alizarin red S (Sigma-Aldrich) was then added to the plates for 15 minutes at room temperature. The residual alizarin red S was removed with water for light microscopy.

Toluidine blue staining [35] to determine the proteoglycan of chondrocytes at day 14 was performed by removing the medium completely and fixing the chondrocyte pellets in neutrally buffered 10% formalin, followed by removal of the formalin using three distilled water-washes. The pellets were then embedded in O.C.T.Compound (Sakura Finetek, Torrance, CA, USA) and sliced with a cryostat (Lecia CM 1950, Lecia Biosysterms, Wetzlar, Germany) at a thickness of 5 μm. The cryostat section was stained with toluidine blue O for 5 min at room temperature. The toluidine blue O was removed with water for light microscopy.

### Preparation of chitosan-coated culture dishes

Chitosan-coated culture dishes were prepared as previously described [30]. Briefly, to prepare a 1% chitosan solution, purified chitosan powder (catalogue #50494 Sigma-Aldrich) was autoclaved in distilled water and dissolved by adding 1 M sterile glacial acetic acid. The bottom of a culture plate was covered with sterile chitosan solution, which was allowed to evaporate to dryness at room temperature in a biological laminar flow hood. This left a thin chitosan acetate film on the surface. The acidity of the surface was then neutralized with sterile 1 N NaOH and the plates were washed several times with sterile distilled water to reach pH = 7.0. They were equilibrated in sterile PBS before being used for cell culture experiments.

### Differentiation of pADSC into Pancreatic Islet-Like Clusters (PILC) on chitosan-coated plates

A one-step procedure for PILC differentiation was modified from a previous study [36]. Briefly, pADSCs were seeded (6 x 10^4^/cm^2^) onto chitosan-coated or regular (or TCPS, tissue culture polystyrene, Corning, NY, USA) plates for one day and the medium was replaced with differentiation medium consisting of serum-free DMEM/F12 in the presence of 10 mM nicotinamide (Sigma N0636), 1 μM exendin-4 (Sigma E7144), 2 nM activin A (ProSpec, Ness Ziona, Israel), 10 nM pentastrin, 123 pM hepatocyte growth factor (Sino Biological Inc., Beijing, China), 1% B27, N2 supplement (Invitrogen, Carlsbad, CA, USA) and 17.5 mM glucose. Plates were cultured in air containing 5% CO_2_ for three days. Unless otherwise indicated, reagents were from Sigma-Aldrich. After three days, the glucose concentration was changed to 5.5 mM for the rest of differentiation period of 15 days with medium changed every three days.

### Immunofluorescence analysis

Immunofluorescence analysis and confocal microscopy procedures were modified from those described previously [22]. Briefly, cells were removed form chitosan coating dishes and then seeded on cover glasses. Then cells were fixed with freshly prepared 4% para-formaldehyde (PFA) for 10 minutes at room temperature (RT), permeabilized with 50% chilled methanol for 20 minutes on ice and blocked with blocking buffer (PBS, 1% FBS and 0.25% Triton X-100) for one hour at RT. Cells were incubated with the primary antibodies against insulin from guinea pig, Pdx-1 or Islet-1 from rabbit (Abcam, CA, USA) overnight at 4°C, washed with PBS and then incubated with the secondary antibodies of goat anti-rabbit IgG DyLight^®^594 ((EpitMics- an Abcam company, CA, USA) or donkey anti-guinea pig IgG DyLight^®^488 (Santa Cruz Biotechnology, Texas, USA) at RT for 1 hour. Slides were washed with PBS and mounted with Ultra Cruz^™^ mounting medium (Santa Cruz Biotechnology, Texas, USA). Cell nuclei were visualized by DAPI (4’, 6-diamidoino-2-phenylindole) (Invitrogen, Carlsbad, CA, USA). Confocal images were obtained with a Leica TCS SP5 II scanning microscope (Leica Microsystems, Wetzlar, Germany) using a HCX PL APO CS 40X/1.25 oil objective with optical slices of ~1 to 2 μm.

Procedures used here for RT-PCR were previously described [29]. To quantify the expression levels of genes associated with pancreatic β-cell differentiation, total RNA was extracted from cells collected on day 0, 3, 6, 9, 12 and 15 using a Total RNA Miniprep Purification Kit (GeneMark, Taichung, Taiwan), digested with DNase I (Ambion, Austin, TX 78744, USA) to remove the contamination by genomic DNA and transcribed to cDNA using a High Capacity cDNA Reverse Transcription kit (Applied Biosystems, Foster City, CA, USA). The real-time quantitative PCR reactions were performed with a CFX96 Real-Time PCR Detection System (Bio-Rad, Richmond, CA, USA) using a DyNAmo Flash SYBR Green Kit (Finnzymes, Espoo, Finland). Running conditions for real-time PCR were initial denaturation at 95°C for 7 min, denaturation at 95°C for 10 s, followed by annealing / extension at 60°C for 30 s for a total of 39 cycles. The primer sequences used were listed in S1 Table. Threshold cycle (Ct) values were obtained and relative gene expression was calculated [27] using the formula (1/2) Ct target gene—Ct β-actin Values were normalized to β-actin levels in the same sample with all measurements in triplicate.

### Glucose challenge and insulin ELISA

Differentiated islet-like clusters at day 9 to 15 were cultured in serum- and insulin-free DMEM/F12 containing 5.5 mM glucose for 24 hr and then challenged with glucose ranging from 5.5 to 28.5 mM for one hour. The medium was collected and analyzed for insulin concentration using an ELISA kit according to the manufacture’s instruction (ALPCO Diagnostics, Windham, NH).

### Withdrawal of the PILC differentiation medium

At day 9, the differentiation medium on the islet-like clusters was replaced with serum –free DMEM/F12 containing N2 and B27. To quantify the expression levels of genes associated with pancreatic β-cell differentiation after withdrawing the differentiation medium, total RNA was extracted from cells collected on day 0, 3, 6 and 9 after withdrawal of differentiation medium. Some of the cells at withdrawal day 9 were cultured in serum- and insulin-free DMEM/F12 containing 5.5 mM glucose for 24 hr and then challenged with glucose ranging from 5.5 to 25 mM for one hour. The medium was collected and analyzed for insulin concentration by using an ELISA kit according to the manufacture’s instruction (ALPCO Diagnostics, Windham, NH).

### Statistical analysis

Statistical significance among different experimental groups was determined by one-way analysis of variance (ANOVA) and Tukey’s test was used to evaluate the differences among the means of different treatments. Results were expressed as mean ± S.E.M. *P* values ≤ 0.05 were considered statistically significant.

## Results

### Isolation and characterization of adipose-derived stem cells from pig dorsal subcutaneous fat depot

**Fig 1:**
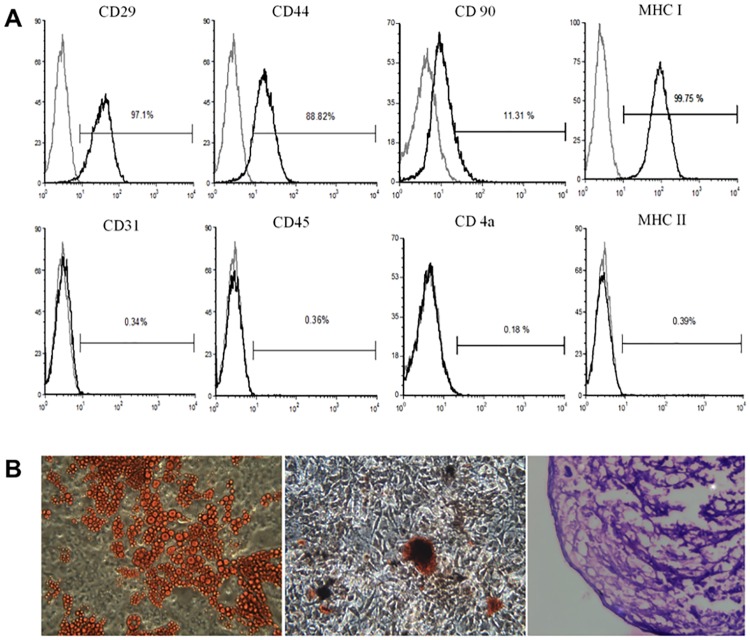
Isolation of multipotent Adipose-Derived Stem Cells (ADSCs) from pig subcutaneous fat depot. (A). Flow cytometric analysis of porcine (p) ADSCs. 1 x 10^6^ cells were analyzed for stem cells markers. Numbers indicate the percentage of stained cells in the population (black) compared with the unstained control (gray). The x-axes represent the relative fluorescence intensity. (B). Multipotency of pADSCs. pADSCs were differentiated into adipocytes (left panel), osteocytes (middle panel) and chondrocytes (right panel) and stained as described in the text. Images were taken at 100 x magnification using phase contrast microscopy.

As shown in Fig 1A, stem cell surface markers, namely CD29, CD44 and MHC I (or HLA I) were detected and negative markers, such as CD 31, CD 45 and MHC II (or HLA II) [3,37] were not detected in our ADSCs isolated from the pig dorsal subcutaneous fat depot, confirming the characteristics of pADSCs. Functional characterization of multipotency also confirms that our pADSCs can be differentiated into adipocytes, osteocytes and chondrocytes (Fig 1B), as stained by specific dyes. These data indicate that we can obtain fully functional pADSCs with characteristics resembling those reported in literature.

### Chitosan-enhanced differentiation of pADSCs into insulin-producing Pancreatic Islet-Like Clusters (PILC)

**Fig 2:**
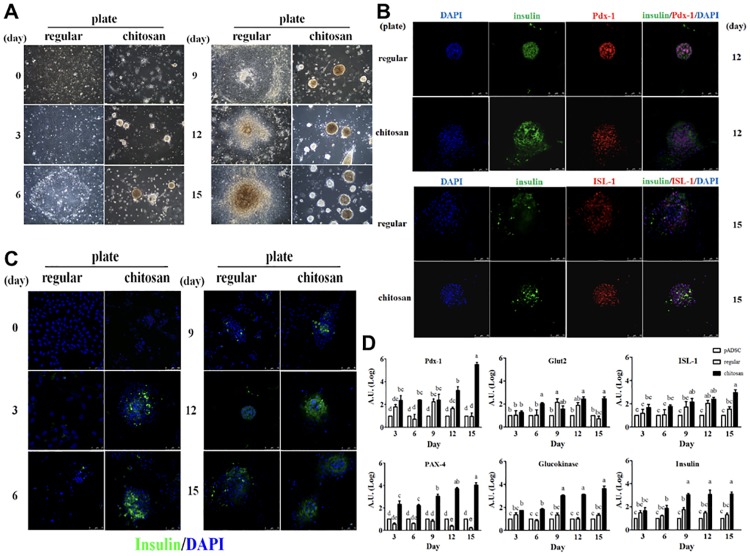
Chitosan enhances differentiation of pADSCs into insulin-producing Pancreatic Islet-Like Clusters (PILC). (A). Representative phase contrast microscopy analysis of the morphology of pADSC-derived PILC. Cells were differentiated for indicated times on regular (left panels) or chitosan-coated (right panels) plates as described in the text. (B). Representative immunofluorescence analysis of β-cell differentiation-associated markers, including Pdx-1, ISL-1 and insulin, in pADSC-derived PILC. PILC grown on regular and chitosan plates on day 12 or 15 were analyzed using antibodies against indicated markers and observed under a confocal microscope. (C). Effects of chitosan on the differential production of insulin by pADSC-derived PILC. The same procedure as in (B) was performed on pADSC differentiation from day 0 to 15. (D). Quantification of the effects of chitosan on the mRNA expression of β-cell differentiation-associated genes in pADSC-derived PILC. Total RNA was extracted from the clusters and analyzed by real-time PCR as described in the text. Values were normalized to β-actin and expressed as mean ± SEM, n = 6. *, *P* < 0.05. (Abbreviations: Pdx-1: pancreatic and duodenal homeobox-1; ISL-1: insulin gene enhancer protein-1; PAX4: pair box gene 4; GLUT2: glucose transporter 2)

Using our one-step procedure, pADSC were induced to form PILC as early as day 3 on chitosan-coated plates. Cells on regular plates did not develop substantial PILC until day 12. Thus, chitosan greatly accelerates the differentiation of pADSC into PILC (Fig 2A). To evaluate the β-cell-associated identity of PILC, we performed immunofluorescence analysis with a confocal microscope, which revealed the co-production of Pdx-1, ISL-1 and insulin (Fig 2B) in both the regular and chitosan-coated plates between days 12 and 15. Chitosan increased the production of insulin on day 3 compared to day 6 on regular plates (Fig 2C).

To further characterize PILC at the molecular level, we measured the mRNA expression profile of β-cell differentiation-related genes, such as *Pdx1*, *Pax4*, *Isl1*, glucokinase, glucose transporter (Glut 2) and insulin, by RT-PCR and found that they were expressed during differentiation (Fig 2D). Each of these PILC-related genes had increased expression during differentiation, especially when cells were cultured on chitosan-coated plates. Specifically, we found that chitosan plates substantially induced the expression of PAX-4, a crucial transcription factor for pancreatic β-cell development [36,38]. The same was found for another essential transcription factor, Pdx-1 and for glucokinase and insulin. One surprising observation was that the expression of the glucose transporter, Glut2 and Pdx-1, suddenly peaked at day 9 and 12 on the regular plate and fell back to the levels of day 3 and 5 as in undifferentiated pADSC. By contrast, chitosan treatment elevated expression of these genes at day 6 and maintained expression through day 15. Taken together, our data indicated that we successfully differentiated pADSC into insulin-producing PILC, with differentiation enhanced by the chitosan matrix.

### Glucose responsiveness of pADSC-derived insulin-secreting clusters

**Fig 3:**
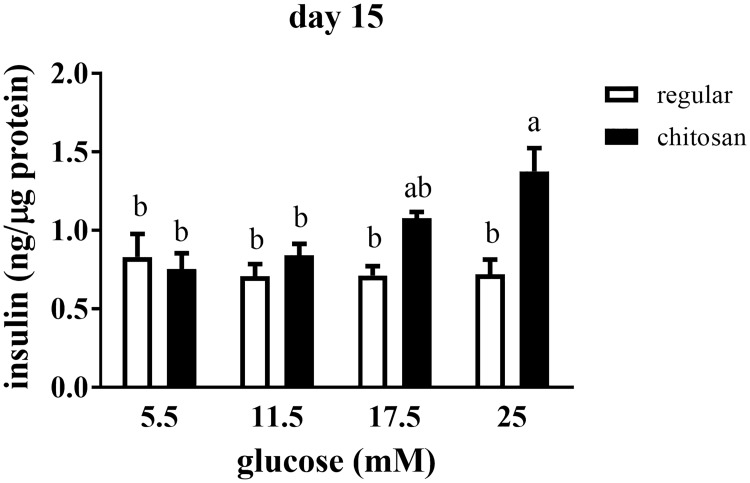
Glucose responsiveness of pADSC-derived insulin-secreting clusters. Quantification of insulin secretion level from PILCs in response to a glucose challenge. PILC from day 15 of differentiation were starved overnight and treated with the indicated concentrations of glucose for one hour. Insulin values were obtained by ELISA analysis as described in the text, normalized against protein concentrations and expressed as mean ± SEM, n = 6. *, *P* < 0.05.

To evaluate the functionality, or glucose responsiveness, of our insulin-producing PILC, we challenged them with different doses of glucose, ranging from basal (5.5 mM) to high (25 mM) concentrations (Fig 3). When PILC were cultured on regular plates a basal level of insulin secretion was detected at day 9 to 15 (from 0.5 to 1.0 ng insulin/μg protein). However, there was no response to increased glucose concentration. Differentiation on chitosan-coated plates enhanced the secretion of insulin by PILC to 1.0 ng / μg protein on day 9 and induced a glucose responsiveness on day 15, with 1.5 ng / μg protein of insulin secretion in response to high (25 mM) glucose levels. These results suggest that the chitosan matrix plays a crucial role in inducing the function of our PILC derived from pADSC.

### The expression of pADSC-derived insulin-secreting clusters after withdrawing the differentiation medium

**Fig 4:**
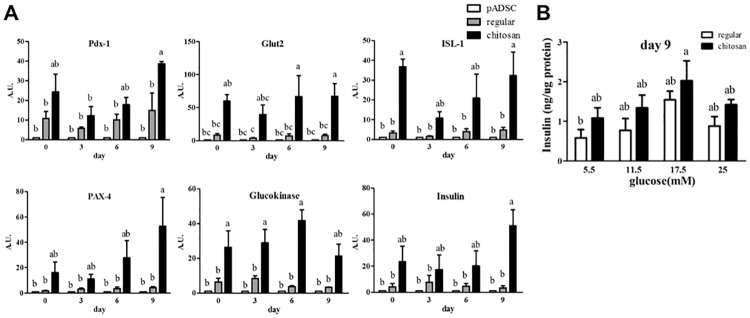
The expression of pADSC-derived insulin-secreting cells and clusters after withdrawing the PILC differentiation medium for 9 days. (A) Quantification of the effects of withdrawing the PILC differentiation medium on the mRNA expression of β-cell differentiation-associated genes in pADSC-derived PILC. The differentiation medium was withdrawn from 0 to 9 days. Total RNA was extracted from the clusters and analyzed by real-time PCR as described in the text. Values were normalized to β-actin and expressed as mean ± SEM, n = 6. *, P < 0.05. (B) Quantification of insulin secretion level from PILCs in response to a glucose challenge on withdrawal day 9. PILCs from day 9 of withdrawal were starved overnight and treated with the indicated concentrations of glucose for one hour. Values were obtained by ELISA analysis as described in the text, normalized against protein concentrations and expressed as mean ± SEM, n = 5. *, P < 0.05.

In order to investigate the function of PILC without the presence of inducing reagents, we withdraw the PILC differentiation medium on differentiation day 9 and measured the mRNA expression profile of β-cell differentiation-related genes, such as Pdx1, Pax4, ISL-1, glucokinase, Glut2 and insulin. Measurements were for 9 days after withdrawal of induction medium. The PILCs kept expressing the β-cell differentiation-related genes and the expression of PILC cultured on chitosan-coated plates was greater than PILC cultured on regular plates. Furthermore, to determine the functionality of our insulin-producing PILC after withdrawing the differentiation medium, we challenged them with different doses of glucose, ranging from basal (5.5 mM) to high (25 mM) concentrations (Fig 4B). The insulin secretion of PILCs on chitosan-coated plates (1.2 to 2 ng/μg protein) was greater than cells on regular plates (0.7 to 1.5 ng/μg protein). However, we surprisingly found that PILC in the continued presence of differentiation medium do not respond to increased glucose or regular plates. Nevertheless, the insulin secretions of both groups had similar patterns, the insulin secretions steadily increased by increasing glucose levels (5.5 mM to 17.5 mM) and sharply decreased in highest glucose level (25 mM). Taken together, the results indicate that the PILCs can spontaneously express β-cells-related genes and produce insulin without stimulated by induction reagents. Moreover, the chitosan matrix can enhance PILCs to express higher β-cell-related genes and to secrete more insulin during withdrawal.

## Discussion

Despite previous efforts to develop stem cell-based approaches for cell therapy and regenerative medicine, a better system using an easily accessible and abundant tissue source with multipotency and a straightforward induction protocol awaits further exploration. We took advantage of subcutaneous ADSC derived from piglets and a differentiation-promoting biomimetic material, chitosan, to generate morphologically and functionally-enhanced PILC. These PILC secreted insulin in response to high glucose levels and have potential to provide a better biomaterial for amelioration of type 1 diabetes.

Pigs have long been recognized as a major animal model for human studies based on their similarities in size, physiology, organ development and disease progression [39]. Of note is that pigs rarely develop insulin resistance, which may be the result of artificial selection for high meat production and, thereby, high pancreatic insulin secretion and tissue insulin sensitivity for energy storage, especially in adipose tissues. Pigs are good models for researching metabolic diseases. We found a high induction potential of pADSC into PILC, but we did not compare the differentiation potential of ADSC from different species. It will, nevertheless, be an interesting topic for future studies, as will studies of the depot-, gender- and age-specific differentiation potential of ADSC into PILC.

Consistent with our previous studies, ADSC-derived from the piglet dorsal subcutaneous fat depot were multipotent, as demonstrated in Fig 1. By modifying the components and concentrations of the differentiation medium (primarily from Timper et al., 2006) for the induction of PILC, we were able to optimize the procedure into one step with medium changes every three days (Fig 2). Glucose is a critical factor for the differentiation of stem cells into β-cells [40]. Moreover, glucose concentrations control the glucose responsiveness of insulin-producing cells and lower concentrations improve colony formation and enhance the differentiation efficiency of stem cells [41]. We used an initial glucose concentration of 17.5 mM that was changed to 5.5 mM at day 3. Nicotinamide not only increases the expression and secretion of Pdx-1, insulin and glucagon in β-cell development [17,42,43], but also prevents the cells from dying or being differentiated into other cell types [44]. We also adapted the concentrations in the differentiation medium from the literature for other β-cell and islet development-associated factors, including activin A [45,46], extendin-4 [17,44,47], hepatocyte growth factor (HGF) [47,48] and pentagastrin [49,50]. These factors contribute to the success of inducing the β-cell differentiation in the present study.

Previous studies [21,22] indicate cell clusters are formed in the process of PILC formation, which led us to propose that a 3D-promoting matrix may facilitate PILC formation and chose chitosan to serve that purpose. Chitosan promotes cell adhesion and differentiation with its unique structure and is extensively used in tissue engineering [51]. Recent studies find that chitosan-induced spheroid formation enhances the multi-lineage differentiation potential of ADSC for chondrogenesis, osteogenesis, neurogenesis, hepatogenesis and cardiomyogenesis [30–32]. The substantially accelerated formation of PILC clusters on chitosan plates on day 2 to 3 (Fig 2A) indicated the value of chitosan. Without chitosan, it usually takes at least 14 days and sometimes even longer for the induction of PILC from hADSC [22]. In our culture system, using the chitosan matrix, pADSC differentiated into β-like cells and secreted insulin in 6 days. In addition to the shortened induction period by chitosan treatment, the functionality of PILC was greatly enhanced and PILC could even produce insulin without stimulation by induction reagents as revealed in Figs 2B, 2C and 4, where the production and expression of pancreatic β-cell markers and genes were confirmed. Our group has tested the expression of NEUROD and NEUROG3 by qRT-PCR analysis and results showed that NEUROD and NEUROG3 were noticeably expressed in day 6 and day 10 of pADSC differentiation into insulin-producing cells (unpublished data). However, following many studies, we choose well-known islet indicators such as Pdx-1, PAX-4, ISL-1, Glucokinase and Glut2 to investigate various differentiation stages of islet-like clusters from pADSC for this current study.

The chitosan matrix plays a crucial role not only in optimizing isletogenesis of pADSC, but also inducing the glucose responsiveness of its terminally differentiated PILC (Fig 3). Liu et al. [32] proposed that chitosan triggers the expression of adhesion molecules on ADSC through Ca^2+^ signaling and, thereby, promotes cell-cell adhesion and spheroid formation. The aggregated cells on chitosan matrix have been shown to have significantly higher “stemness” marker genes, Oct4, Nanog and Sox10, which may help their multipotent differentiation abilities [52]. Therefore, the chitosan matrix can be an important first step to isolate the spheroid-forming subpopulation, which has more multipotent differentiation potential. Some studies report that the substrates in cultured pancreatic β-cells affect the performance and morphology of β-cells and that aggregated β-cells have better performance than monolayer β-cells [53]. Previous study has been shown that native pig islets can produce 1200 μU insulin per mg protein which is approximately 40 ng insulin/ μg proteins [54]. In our current study, PILC, differentiated on chitosan-coated plates, secreted approximately 1 ng insulin per μg protein on day 9. Upon glucose-stimulated insulin secretion (GSIS) test with high (25 mM) glucose levels, chitosan-coated plate-differentiated PILC secreted about 1.5 ng insulin per μg protein on day 15 (Fig 4B). The ability of insulin secreting ability is much improved over previous study [17].

The matrix influences the growth and differentiation of adult stem cells, which has been demonstrated in many studies, but not for islet differentiation. A collagen/hyaluronic scaffold produces three-dimensional structures that enhance the differentiation of insulin producing cells [55]. In our study, we also found similar effect in chitosan matrix, and only needed 9 days to differentiate into insulin producing cells with chitosan matrix in comparison to 18 differentiating days of Khorsandi's group [55]. We suggest chitosan is an efficient matrix for islet differentiation. Other polymeric biomaterials can be used as a matrix to support stem cells. PMVE-alt-MA, inexpensive polymer, was screened out from 91 polymers by polymer array and the results showed PMVE-alt-MA can efficiently support ESC long term self-renew and proliferation [56]. However, there are few comparative studies regarding various matrices and the mechanism of the interaction between matrix and stem cells is essentially unknown.

The chitosan matrix is highly recommended to apply to cell therapy for diabetics. Over all, our system can serve as a unique model to study β-cell maturation and the mechanisms underlying the regulation of glucose-induced insulin secretion. Sneddon *et al*. [57] have a procedure, by which embryonic stem cells can be massively expanded to a specific progenitor, for instance, cells that can be differentiated into β-cells by being co-cultured with primary mesenchymal cells derived from the pancreas. In addition, mesenchymal stem cells can be proliferated and maintain their differentiation potential using precondition treatments [58]. By adapting these protocols, we expect that in the future, significant amounts of uniform PILC with high glucose responsiveness and insulin secretion capability can be generated from adipose tissues. The therapeutic potential of PILC, derived from pADSC, for Type 1 diabetes may be potentiated by encapsulation to overcome the species immune barrier [4] during xeno- and allo-transplantation.

## Conclusion

Our current study successfully developed a chitosan matrix-enhanced one-step procedure to differentiate ADSC from pig dorsal subcutaneous fat depots into high glucose-responsive insulin-secreting clusters, which have potential to ameliorate Type 1 diabetes.

## Supporting information

S1 Table Primers used for Q-PCR. (DOC)
