# Supplementary material for: Chitosan-assisted differentiation of porcine adipose tissue-derived stem cells into glucose-responsive insulin-secreting clusters
Source: PLoS One. 2017 Mar 2;12(3):e0172922. doi: 10.1371/journal.pone.0172922 (PMC5333835; doi:10.1371/journal.pone.0172922)
Supplement: S1 Table — (DOC) [file pone.0172922.s001.doc]

Supplemental Table 1. Primers used for Q-PCR

|  |  | |  |
| --- | --- | --- | --- |
| Genes |  | Sequence | GenBank accession number |
| Pdx-1 | Forward | 5'- CCG TGG ATG AAG TCT ACC -3' | NM_001141984.1 |
|  | Reverse | 5'-GCCTAGAGATGTATTTGTTGAAA-3' |  |
|  |  |  |  |
| PAX-4 | Forward | 5'-GCACCCAGGACAAGACTC-3' | LOC100523579 |
|  | Reverse | 5'-GGAGCCTCAGAGCACTA-3' |  |
|  |  |  |  |
| Glucokinase | Forward | 5'-ATGAAGACCGCCGATGTG-3' | XM_003484067.1 |
|  | Reverse | 5'-GTCATACTCCAGCAGGAACTC-3' |  |
|  |  |  |  |
| Glut2 | Forward | 5'-TGTGAGTATGACAGCCATATTCCT-3' | NM_001097417.1 |
|  | Reverse | 5'-TGAAATTGCGGGTCCAGTTG-3' |  |
|  |  |  |  |
| ISL-1 | Forward | 5'-AAGGAGGACCGGGCTCTAAT-3' | NC_010458.3 |
|  | Reverse | 5'-GGACTGGCTACCATGCTGTT-3' |  |
|  |  |  |  |
| Insulin | Forward | 5'-CTTCTTCTACACGCCCAAGG-3' | NW_003540879.1 |
|  | Reverse | 5'-CACGATGCCACGCTTCTC-3' |  |
|  |  |  |  |
| β-Actin | Forward | 5'-GCCAGGTCATCACCATCGG-3' | AY550069 |
|  | Reverse | 5'-GTAGAGGTCCTTGCGGATGTC-3' |  |
